# Investigating the quality of hemovigilance process using the first two steps of Six Sigma model: a cross-sectional study

**DOI:** 10.1186/s12913-023-10113-6

**Published:** 2023-10-27

**Authors:** Fatemeh Molaahmadi-Hassanabadi, Mohammad Hossein Mehrolhassani, Rohaneh Rahimisadegh

**Affiliations:** 1 Health Services Management Research Center, Institute for Futures Studies in Health, Kerman University of Medical Sciences, Kerman, Iran; 2 Department of Health Management Policy and Economics, Faculty of Management and Medical Information Sciences, Kerman University of Medical Sciences, Kerman, Iran

**Keywords:** Patient safety, Quality improvement, Six sigma, Hemovigilance, Blood transfusion errors, Blood bank, Hospital

## Abstract

**Background and purpose:**

Hemovigilance is a set of monitoring methods that covers the blood transfusion chain, from collecting blood and blood products to monitoring the blood recipients. To this end, any error in this process can have serious and irreparable consequences for patients. The present study aimed to investigate the quality of hemovigilance process in Iran, using the first two steps of Six Sigma model.

**Methods:**

This was a quantitative cross-sectional study that was conducted over 6 months (from August 20, 2021, to February 20, 2022) at Afzalipour Hospital in Iran, using the first two steps of Six Sigma model. The study population comprised of all inpatients who needed blood or blood product transfusion in various departments of Afzalipour Hospital, among whom 477 patients were selected via stratified sampling in three shifts (morning, evening, and night). The datasheet was used to record errors in the three shifts. This research was conducted, using the DMAIC cycle’s “define” and “measure” steps.

**Results:**

In the define step, the hemovigilance process at Afzalipour Hospital was divided into two categories of normal process and emergency process. Each of these processes consists of several sub-processes, including “phlebotomy,“ “requesting blood and blood products from the department,“ “preparation of application by the blood bank,“ " sending a request from the blood bank to the blood transfusion center,“ “transfusing blood and blood products,“ and “returning the blood and blood products to the blood bank and waste disposal.“ In the measure step, the quality of hemovigilance process was evaluated based on sub-processes and labels at morning, evening and night shifts. The sub-process of sending a request from the blood bank to the blood transfusion center had the highest error rate with a sigma level of 1.5. Also, the evening and night shifts had a sigma level of 1.875, and the clinical and registration labels had a sigma level of 1.875. The overall sigma level of hemovigilance process was calculated to be 2.

**Conclusion:**

The results of this study showed that the quality of hemovigilance process at Afzalipour Hospital was poor. By employing the first two steps of Six Sigma method, we identified the existing errors in the hemovigilance process of Afzalipour hospital in order to assist hospital managers to take the necessary measures to improve this process.

**Supplementary Information:**

The online version contains supplementary material available at 10.1186/s12913-023-10113-6.

## Introduction

Patient safety, as one of the healthcare priorities, is defined as “reducing the risk of healthcare-related harm to minimum”. Despite efforts being made to improve patient safety in healthcare, unsafe clinical care and services continue to occur, resulting in approximately 15% of total hospital costs in addition to patient harm [1, 2]. The medical diagnosis laboratory is one of the most important departments in every hospital, as more than 70% of crucial medical diagnosis decisions rely on the outcomes of laboratory tests. Errors in this department’s work processes can result in the wrong or delayed treatment, which consequently endanger patient safety [3]. This is while most errors are preventable [4].

Many processes are performed in medical diagnosis laboratories, including hemovigilance process [5]. The term hemovigilance is a combination of the Greek word “hema = blood” and Latin word “vigilance = alertness”. Hemovigilance is a collection of monitoring methods that covers the blood transfusion chain (from blood or blood product collection to monitoring the recipient) for the purpose of data collection and evaluation [6]. Blood transfusion is a complex process consisting of several sub-processes [7]. It involves various individuals, including physicians, nurses, blood bank personnel, hospital porters, hospital drivers, and personnel of blood transfusion center [8]. Also, any error in each of the blood transfusion sub-processes can have severe and irreparable consequences for patients [9].

The Serious Hazards of Transfusion (SHOT) report in 2018 showed that 84% of error reports were related to human errors, and only 10% were unavoidable [4]. Errors can occur at any stage of blood transfusion process, including improper collection and labeling of blood samples, improper transportation of blood and blood products from the blood transfusion center to the hospital blood bank and sending them to the departments, improper identification of blood group and crossmatching, and failure to check the patient’s information before transfusing the blood. These errors occur as a result of not following the hemovigilance procedure’s guidelines and standards [6, 7].

To prevent the occurrence of errors and improve the quality of blood transfusion services, it is necessary to employ a variety of management approaches and techniques that help to predict the occurrence of errors and implement the required safety measures [10]. The Six Sigma method is an effective method for identifying errors and improving processes. Six Sigma has been regarded as a systematic and effective method for promoting and enhancing the quality of healthcare services, controlling costs, and improving patient safety [11]. Six Sigma is implemented with the assistance of a standard DMAIC cycle and special tools, such as fishbone diagram and SIPOC (Suppliers, Inputs, Processes, Outputs, and Customers). The steps of DMAIC cycle include define, measure, analyze, improve and control [12].

Unfortunately, despite the importance of patient safety in the hemovigilance process in hospitals, over the past few years, errors and challenges in this process have resulted in unwanted and undesirable patient complications. This study is one of the few studies conducted in Iran to employ the Six Sigma methodology to examine the quality of hemovigilance process. Considering the importance of hemovigilance process in patient safety, this study was conducted to investigate the quality of hemovigilance process at Afzalipour Hospital, using the first two steps of Six Sigma method.

## Method

### Study design

This was a quantitative cross-sectional study that was conducted to investigate the quality of hemovigilance process over a period of 6 months from August 20, 2021, to February 20, 2022 at Afzalipour Hospital, Kerman, using the first two steps of Six Sigma model. The Afzalipour Hospital, which is located in the center of Kerman province (the southeast of Iran), has a super-specialized oncology, transplant, women, children, surgical and medical wards that offer various services to the members of public. This hospital has 700 active beds and an annual average of 160,000 admissions.

### The population and sample

The study population consisted of all inpatients who required blood or blood product transfusion in different departments of Afzalipour Hospital. Using the stratified sampling method and following formula, and assuming 10% error rate, the number of samples in each process was calculated to be 90 patients, and in total processes, the sample size was determined to be 477 patients.

$${n}_{ss}={\left(\frac{1}{\delta}\left(\frac{{\sigma}_{ss}}{\mu}100\right)4/50\right)}^{2}$$

$$\delta =2/5$$

13 $${CV}_{ss} =\left(\frac{{\sigma}_{ss}}{\mu}100\right)=5$$

### Six Sigma model

The Six Sigma approach, which is used to identify and eliminate process errors that occur within DMAIC cycle, consists of two parts. The first part is related to “understanding and identifying problems”, and has three initial steps of define, measure, and analyze. The second part of the cycle includes “problem-solving”, and consists of two steps of “improve” and “control” [12]. This study was conducted based on the two steps of “define” and “measure” in the first part of DMAIC cycle.

### Study implementation steps

#### Define step

To obtain a comprehensive definition of hemovigilance process at this step, the national guidelines of blood transfusion center, and also the internal regulations and guidelines of Afzalipour Hospital were reviewed. After attending the hospital departments and blood bank, the researchers directly observed the hemovigilance process in the hospital, and also conducted four semi-structured interviews with the head of blood bank unit, the head of blood transfusion center, the educational supervisor, and the nursing hemovigilance officer to collect information and complete the observations (Additional file 1). A written informed consent was taken from all participants.

**Fig. 1:**
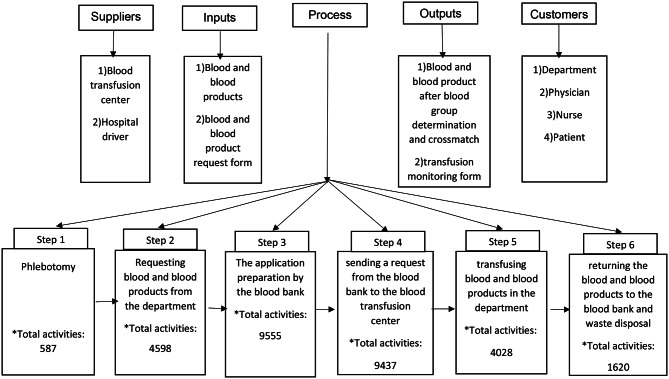
Suppliers, inputs, process, outputs and customers diagram of the Hemovigilance process in Afzalipour hospital *Each step included both normal and emergency activities

All steps of hemovigilance process were drawn by the BPMN.2 standard in the Visual Paradigm software, using the information obtained from the previous steps with the research team’s opinion. The SIPOC tool was also used at this step to create a process map and identify suppliers, inputs, outputs, and customers in the hemovigilance process (Fig. 1).

Finally, after analyzing the interviews and observations, and also reviewing the regulations and instructions (document review), the standards, actions, and activities in each process, as well as any potential errors, were determined. During this step, the clinical activities performed by nurses, midwives, physicians, phlebotomists, and blood bank personnel were considered as clinical labels, and activities related to the equipment in the blood bank and hospital departments were regarded as equipment labels. The activities associated with completing the information on the blood transfusion form or registering information in the hospital information system (HIS) were also considered as registration label, and activities performed by the hospital driver and porter were regarded as a support label. Accordingly, the analysis of findings was categorized.

#### Measure step

This step is related to measuring the error rate and determining the correct measurement scale. To this end, the error rate was determined by investigating the hemovigilance process for inpatients who required blood or blood product transfusions, and its compliance with the standards established in the define step.

The data collection tool in this step included the datasheet form (laboratory non-conformity forms in the hospital), which was developed by reviewing internal guidelines and documentation, and also conducting interviews with experts. The validity of this tool was confirmed by the manager of blood transfusion center, educational supervisor, blood bank manager and the research team (Additional file 1). The researcher gathered data through direct observation by visiting the blood bank and various hospital departments in three morning, evening and night shifts, while using a datasheet form. After collecting the data, they were analyzed by Microsoft Excel, and the error rate was calculated.

The sigma level was determined using the defects per million opportunities (DPMO) and defects per unit (DPU) The DPMO scale has been developed for times when DPU is normalized in terms of the complexity of operation and the way it is performed. It also means how much error occurs in that process for every one million times the process is carried out. Using the following formulas and measurements, DPU was determined by dividing the number of defects in each unit and sample size. The resulting DPU was used to calculate the amount of DPMO. The result was multiplied by one million, then divided by the mean number of defect opportunities. All hemovigilance processes collected on the datasheet are considered total opportunities for defects to occur. Finally, all the formula-derived information was entered into Tables 1 and 2, and 3.

$${\rm{DPU}} = \frac{{number\,of\,defects}}{{sample\,size}}$$

14 $${\rm{DPMO}} = \frac{{DPU * 1000000}}{{mean\,of\,defect\,opportunities}}$$

Based on the above formulas, DPU and DPMO for sub-process of “application preparation by the blood bank and sending it to the department” were calculated as follows:

$${\rm{DPU}} = \frac{{3074}}{{90}} = 34.15$$

$${\rm{DPMO}} = \frac{{34.15 * 1000000}}{{106.16}} = 321684.2$$

Finally, based on the obtained DPM, the sigma level of the “application preparation by the blood bank and sending it to the department” was determined to be 2 [15].

## Results

### Define step

The hemovigilance process at Afzalipour Hospital is generally divided into two categories of normal process and emergency process based on the severity of patient’s need for blood or blood products and the physician’s diagnosis. Each of these processes consists of several sub-processes, including “phlebotomy,“ “requesting blood and blood products from the department,“ “the application preparation by the blood bank and sending it to the department,“ “sending a request from the blood bank to the blood transfusion center, and delivering the required blood bags and blood products,“ transfusing blood and blood products in the department,“ and “returning the bloods and blood products to the blood bank and disposing them.“

### Measure step

At this step, we measured the errors, and each error had a series of consequences. The errors were divided into four categories based on their consequences: (1) Errors, which had long-term consequences without having a immediate effect within the hospital environment, often occurred after patient discharge from the hospital. These errors were outside our control and we could not report such consequences. (2) Errors such as registration errors that did not result in serious consequences. (3) Errors that had serious clinical consequences and were observable. (4) Errors that could have immediate effect and consequences within the hospital environment, but we were not aware of such consequences.

Since many errors overlap between sub-processes, shifts and labels, only the serious consequences of most frequent errors that occurred within sub-processes were discussed in this study. Additionally, the number of errors that occurred was identified for the most important actions and activities.

The sub-process of sending a request from the blood bank to blood transfusion center and delivering the required blood bags and blood products had the highest error rate with a sigma level of 1.5, while the process of returning the blood and blood products to the blood bank and waste disposal had the lowest error rate with a sigma level of 2.75. The overall sigma level of hemovigilance process was 2 (Table 1).

During the sub-process of sending a request from the blood bank to blood transfusion center and delivering the required blood bags and blood products, activities such as “delivery of thermometer and standard cold box for transporting blood or blood products by the blood bank personnel to the hospital driver (n = 90),“ “placing bloods or blood products received from the blood transfusion center in the refrigerator in order of expiration date (n = 89),“ " attending the blood transfusion center with a specialized vehicle (n = 89),“ and “checking of blood or blood products and printed receipts by the blood bank personnel” accounted for the most errors, whereas “the transportation method of blood and blood products by the hospital driver” accounted for the least errors.

The errors in these actions and activities led to the incomplete preservation of cold chain during the transportation of blood and blood products. Due to the lack of thermometer in the cold box, it was not possible to measure the temperature of blood bags and blood products, which could have resulted in temperature exceeding the standard limits.

In the phlebotomy sub-process, most errors were related to “obtaining informed consent from patient or patient’s trusted companion (n = 27)” and " identification of patient by the nurse by asking patient if he is conscious, and checking the information of wristband and file number if he is unconscious.“ Also, the least errors were related to the “method of phlebotomy.“

The failure to obtain informed consent from patients or obtaining consent for transfusing blood or blood product by the nurse using an incorrect method resulted in patient complaints and dissatisfaction.

The activities that demonstrated the most errors in the sub-process of “application preparation by the blood bank and sending it to the department, included “registering the hospital porter’s profile in the order book for sending blood or blood products to the department,“ “using the standard crossmatch label after preparing the blood bag or blood product,“ “using the slide test instead of tube test when determining blood group (n = 59),“ and “back type and cell type processes when determining blood group (n = 75),“, Also, the least number of errors were associated with “checking the request received from the department by blood bank personnel.“

An error in determining blood group by the blood bank personnel led to a wrong blood group being determined, which ultimately resulted in a reaction during blood transfusion to patient.

In the sub-process of transfusing blood and blood products, the activities that accounted for the most errors included; “mandatory presence of shift manager as a witness at the patient’s bedside”, “controlling the patient’s consent form for a blood transfusion by the nurse”, “identifying patient by the nurse by asking the patient if he is conscious and checking the information of wristband and file number if he is unconscious (n = 53)”, “matching the patient’s identity with the information of blood profile form sent to the patient and the blood or product request form by two nurses(one controlling the activity and the other carrying out the transfusion), (n = 52)”, “checking the vital signs of patient and recording it in the transfusion monitoring form before the transfusion by the nurse administering the transfusion (n = 47)”, “being at the patient’s bedside and monitoring the patient in the first 15 minutes of transfusion (n = 40)” and “providing complete explanations to patient by the physician and the nurse about the advantages and possible side effects of transfusion,“. Meanwhile, the least number of errors was observed in the activity of “completing the transfusion reaction reporting form (TRRF).“

The errors in these actions and activities led to the death of an infant and an elderly patient during blood transfusion at the hospital. These two individuals died due to their weakened immune system, other clinical problems, and failure to follow hemovigilance protocol by nurses.

Most errors in the sub-process of requesting blood and blood products from the department were associated with “completing the blood and blood product request form by the phlebotomist and the physician” and “completing the blood sample label at the patient’s bedside (n = 43),“ while the least errors were associated with “registering the request in HIS by the nurse.“

The incomplete filling of blood and blood products request form by the phlebotomist and physician, and its submission to the blood bank, caused a delay in the blood bank testing process to the extent that the blood bank personnel had to complete this form instead of starting the relevant tests.

“Completing the form of non-use of blood and blood products monthly by blood bank personnel” was the most error-prone activity, while “labeling the returned blood bags and blood products on another sheet” and “sending the form or to the blood transfusion center” were the activities with the least errors in the sub-process of returning the bloods and blood products to the blood bank and waste disposal.

**Table 1:** The evaluation results of the quality of hemovigilance process

Sub-processes	sample size	number of defects (percentage of all defects)	number of defect opportunities	average of defect opportunities	DPU	DPMO	sigma level
sending a request from the blood bank to the blood transfusion center and delivering the required blood bags and blood products	90	4656 (47.35%)	9437	104.85	51.73	493377.1	≈ 1.5
Phlebotomy	27*	209 (2.12%)	587	21.74	7.74	356047.7	≈ 1.875
Application preparation by the blood bank and sending it to the department	90	3074 (31.26%)	9555	106.16	34.15	321684.2	≈ 2
Transfusing blood and blood products in department	90	1001 (10.18%)	4028	44.75	11.12	248491.6	≈ 2.125
Requesting blood and blood products from the department	90	712 (7.24%)	4598	51.08	7.91	154849.9	≈ 2.5
Returning the blood and blood products to the blood bank and waste disposal	90	180 (1.83%)	1620	18	2	111111.1	≈ 2.75
Total	477	9832 (100%)	29,825	62.52	20.61	329654.5	≈ 2

*: Since phlebotomy is mostly done in the morning shift, and also as phlebotomy is considered an emergency in the evening and night shifts, the sample size of the phlebotomy sub-process was less than the other sub-processes

In Table 2, the quality of normal and emergency hemovigilance processes was measured in three shifts (morning, evening, and night). The evening and night shifts had the highest error rate with a sigma level of 1.875, while the morning shift had the lowest error rate with a sigma level of 2. Among the hospital’s departments and blood bank, the blood bank experienced the most errors.

The most errors in the night shift were related to “completing the patient’s blood sample label information,“ “delivery of the thermometer and standard cold box for transporting blood or blood products by the blood bank personnel to the hospital driver,“ and " using the standard crossmatch label after preparing the blood or blood product,“ " attending the blood transfusion center with a specialized vehicle,“ " placing blood or blood products received from the blood transfusion center in the refrigerator in order of expiration date”, “using the slide test instead of tube test when determining the blood group,“ and “checking the blood or blood product and printed receipt by the blood bank personnel.“ The least errors were associated with “matching the blood or blood product request in the HIS with the request form by the blood bank personnel,“ “matching the patient’s profile on the sample to the profile on the blood and blood product request form by the blood bank personnel,“ “method of transporting the platelet product by the hospital driver,“ “checking the patient’s vital signs during the transfusion of each product and recording it in the transfusion monitoring form by the nurse administering the transfusion,“ and “the method of phlebotomy by the phlebotomist.“

In the evening shift, the most errors were related to " identifying patient by the nurse by asking the patient if he is conscious and checking the information of wristband and file number if he is unconscious “, “matching the patient’s identity with the information of the blood profile form sent to the patient and blood or blood product request form by two nurses (one controlling the activity and the other carrying out the transfusion),“ and “controlling the patient’s consent form for a blood transfusion by the nurse.“ Also, “matching the requested blood or blood product in the HIS with the request form by the blood bank personnel”, “proper storage of blood or blood product in the department until the time of transfusion by the nurse " and “how to transport blood and blood products using a cold box by the driver” accounted for the fewest errors during the evening shift.

Most errors occurred during the morning shift in the activities of the “delivery of thermometer and standard cold box for transporting blood or blood products by the blood bank personnel to the hospital driver,“ " using the standard crossmatch label after preparing the blood bag or blood product,“ and " identifying patient by the nurse by asking the patient if he is conscious and checking the information of wristband and file number if he is unconscious.“ Also, “blood transfusion by only the nurse administering the transfusion,“ “recording the information on the sample label,” “notifying the department by blood bank personnel if the information in the HIS does not match the written request,“ and “monitoring the patient’s vital signs during blood transfusion and recording them on the transfusion monitoring form” accounted for the least errors.

**Table 2:** The evaluation results of the quality of hemovigilance process in three shifts

sub-process location	sub-process	shift	sample size	number of defects (percentage of all defects)	number of defect opportunities	average of defect opportunities	average of defect opportunities	DPMO	sigma level
blood bank	sending a request from the blood bank to the blood transfusion center and delivering the required blood bags and blood products	E	30	1772 (18.02%)	3169	105.63	59.06	559166.9	≈ 1.25
		M	30	1293 (13.15%)	3198	106.6	43.1	404315.2	≈ 1.75
		N	29	1546 (15.72%)	2945	101.55	53.31	524957.6	≈ 1.5
	sending a request from the blood bank to the blood transfusion center and delivering the required blood bags and blood products (emergency)	N	1	45 (0.45%)	125	125	45	360000	≈ 1.875
	application preparation by the blood bank and sending it to the department	E	30	946 (9.62%)	3125	104.16	31.53	302707.4	≈ 2
		M	27	819 (8.32%)	2809	104.03	30.33	291550.5	≈ 2
		N	28	1127 (11.46%)	3040	108.57	40.25	370728.6	≈ 1.875
	application preparation by the blood bank and sending it to the department (emergency)	M	3	78 (0.79%)	359	119.66	26	217282.3	≈ 2. 25
		N	2	104 (1.05%)	222	111	52	468468.5	≈ 1.625
	returning the blood and blood products to the blood bank and waste disposal	M + N + E *	90	180 (1.83%)	1620	18	2	111111.1	≈ 2.75
department	requesting blood and blood products from the department	E	30	252 (2.56%)	1527	50.9	8.4	165029.5	≈ 2.5
		M	27	171 (1.73%)	1364	50.51	6.33	125366.6	≈ 2.625
		N	30	246 (2.50%)	1511	50.36	8.2	162806.1	≈ 2.5
	requesting blood and blood products from the department (emergency)	M	3	43 (0.43%)	196	65.33	14.33	219387.8	≈ 2.25
	transfusing blood and blood products in department	E	53	645 (6.56%)	2413	45.52	12.16	267153.3	≈ 2.125
		M	8	94 (0.95%)	355	44.37	11.75	264818.6	≈ 2.125
		N	29	264 (2.68%)	1260	43.44	9.03	207872.9	≈ 2.375
	phlebotomy	M	24	187 (1.90%)	522	21.75	7.79	358237.6	≈ 1.75
		N	3	22 (0.22%)	65	21.66	7.33	338461.5	≈ 1.875
blood bank and departments	total processes	Total evening shift	143	3615 (36.76%)	10181	71.19	25.27	354965.6	≈ 1.875
		Total morning shift	122	2658 (27.03%)	8795	72.09	21.78	302122.3	≈ 2
		Total night shift	122	3352 (34.09%)	9139	74.9	27.47	366755.7	≈ 1.875
		total shift	477	9832 (100%)	29825	62.52	20.61	329654.5	≈ 2

*M + N + E: Since blood reports and returned products were not registered separately by shift, all three shifts were considered together

M = morning

N = night

E = evening

In Table 3, the activities of hemovigilance process were separated based on clinical, support, equipment, and registration labels, so that the clinical and registration labels accounted for the most errors with a sigma level of 1.875 and the support label accounted for the least errors with a sigma level of 2.5.

The most errors identified in activities involving clinical labels included; “placing blood and blood products received from the blood transfusion center in the refrigerator in order of expiration date”, “checking the temperature of thermometer inside the cold box”, “checking the blood or blood products received from the blood transfusion center by the blood bank personnel”, and “using the standard crossmatch label after preparing the bloods or blood products”. Also, the least errors were identified in activities such as"the timing of plasma transfusion”, “matching the requested blood or blood product in the HIS with the request form by the blood bank personnel”, “notifying the department by blood bank personnel if the information in the HIS does not match with the written request”, “blood transfusion by only the nurse administering the transfusion”, “checking the vital signs during blood and blood product transfusion and recording them on the transfusion monitoring form by the nurse administering the transfusion”, “phlebotomy”, “crossmatch test”, and “placing platelets in the shaker as soon as they arrive from the blood transfusion center by blood bank personnel.“

Activities involving support labels that had the most errors included; “visual inspection of blood bags by the hospital driver during delivery from the blood transfusion center” and “matching the blood bags and blood products received with the list of products sent from the blood transfusion center by the driver.“ In contrast, “the driver transporting only one type of blood product in the cold box,“ “the driver transporting platelet product,“ and “the hospital porter sending application forms and blood samples to the blood bank” accounted for the least errors.

Activities involving the equipment labels, including “delivery of thermometer and standard cold box for transporting blood or blood products by blood bank personnel to the hospital driver,“ were evaluated as having the most errors, while the activities such as “delivery of necessary transportation equipment (ice pack, absorbent cloth, bubble wrap, and plastic envelope) from blood bank personnel to the driver” were evaluated as having the least errors.

The most errors in activities involving registration labels included; “completing the form of unused blood and blood products on a monthly basis,“ “registering the time of blood sample arrival to the blood bank and obtaining the signature of person delivering the blood or blood product in the crossmatch booklet by the blood bank personnel,“ “completing a printed receipt by the blood transfusion center,“ “recording the time of blood product arrival from the blood transfusion center in the blood and blood product request form by the hospital representative,“ and “recording the recommended time or speed of blood transfusion by the doctor in the blood and blood product request form.“ Meanwhile, the least errors were related to activities, including “recording the phlebotomy time and the name of phlebotomist on the blood sample label,“ “recording the patient’s medical history and the reason for blood or blood product transfusion by the doctor in the blood and blood product request form,“ “completing the platelet apheresis request form by the requesting physician,“ “completing the irradiated blood and blood product request form by the attending physician” and “completing the blood and blood product specification form sent from the blood bank by the blood bank personnel”.

**Table 3:** The evaluation results of the quality of hemovigilance process based on labels

sub-process	label	sample size	number of defects (percentage of all defects)	number of defect opportunities	average of defect opportunities	DPU	DPMO	sigma level
sending a request from the blood bank to the blood transfusion center and delivering the required blood bags and blood products	clinical	89	462 (4.71%)	710	7.97	5.19	650704.2	≈ 1.125
	support	89	467 (4.76%)	1641	18.43	5.24	284582.6	≈ 2.125
	equipment	89	372 (3.79%)	1157	13	4.17	321521.2	≈ 2
	registration	89	3310 (33.80%)	5804	65.21	37.19	570296.3	≈ 1.375
sending a request from the blood bank to the blood transfusion center and delivering the required blood bags and blood products (emergency)	clinical	1	0	6	6	0	0	0
	support	1	0	14	14	0	0	0
	equipment	1	4 (0.04%)	13	13	4	307692.3	≈ 2
	registration	1	41 (0.41%)	92	92	41	445652.2	≈ 1.625
application preparation by the blood bank and sending it to the department	clinical	85	1235 (12.61%)	3414	40.16	14.52	361553.8	≈ 1.875
	support	85	0	875	10.29	0	0	0
	registration	85	1621 (16.55%)	4630	54.47	19.07	350,101	≈ 1.875
application preparation by the blood bank and sending it to the department (emergency)	clinical	5	60 (0.61%)	226	45.2	12	265486.7	≈ 2.125
	support	5	6 (0.06%)	66	13.2	1.2	90909.09	≈ 2.875
	registration	5	114 (1.16%)	285	57	22.8	400,000	≈ 1.75
returning the blood and blood products to the blood bank and waste disposal	clinical	90	0	90	1	0	0	0
	support	90	0	90	1	0	0	0
	registration	90	180 (1.83%)	1440	16	2	125,000	≈ 2.625
requesting blood and blood products from the department	clinical	87	0	87	1	0	0	0
	support	87	14 (0.14%)	354	4.06	0.16	39548.02	≈ 3.25
	registration	87	655 (6.68%)	3961	45.52	7.52	165362.3	≈ 2.5
requesting blood and blood products from the department (emergency)	clinical	3	0	10	3.33	0	0	0
	support	3	0	12	4	0	0	0
	registration	3	43 (0.43%)	174	58	14.33	247126.4	≈ 2.125
transfusing blood and blood products in department	clinical	90	862 (8.80%)	2778	30.86	9.57	310110.2	≈ 2
	support	90	28 (0.28%)	90	1	0.31	311111.1	≈ 2
	registration	90	109 (1.11%)	1152	12.8	1.21	94618.05	≈ 2.875
phlebotomy	clinical	27	181 (1.84%)	371	13.74	6.7	487870.6	≈ 1.5
	registration	27	28 (0.28%)	216	8	1.03	129629.6	≈ 2.625
total processes	total clinical	477	2800 (28.59%)	7692	16.12	5.87	364143.9	≈ 1.875
	total support	450	515 (5.25%)	3142	8.72	1.14	131126.7	≈ 2.5
	total equipment	90	376 (3.83%)	1170	13	4.17	321367.5	≈ 2
	total registration	477	6101 (62.30%)	17,754	37.22	12.79	343632.5	≈ 1.875
	total labels	1494	9792 (100%)	29,758	19.91	6.55	328980.4	≈ 2

## Discussion

This study was conducted to examine the quality of hemovigilance process in the Afzalipour Hospital in Kerman, Iran, using the first two steps of Six Sigma method, and also to develop strategies for identifying and reducing error rates.

According to the results of two define and measure steps, the hemovigilance process included several sub-processes such as phlebotomy, requesting blood and blood products from the department, preparing application by the blood bank and sending it to the department, sending a request from the blood bank to the blood transfusion center and delivering the required bloods and blood products, transfusing blood and blood products, and returning the blood and blood products to the blood bank and waste disposal. Errors in three morning, evening and night shifts were observed and recorded in different departments as well as hospital’s blood bank.

According to the obtained sigma level, clinical label activities and actions, evening and night shifts, and hospital blood bank accounted for the highest error rates. A study conducted by Kaur et al. in one of India’s hospitals revealed that the laboratory was responsible for 48% of all errors, while the departments were responsible for 46% of all errors. Also, the evening shift accounted for 49.4% of all errors while the night shift accounted for the most errors (30.1%). In addition, the lowest error rate (20.5%) was related to the morning shift. The most common causes of errors in this study included interruptions, distraction, and heavy workload [16]. According to Sidhu’s research, most errors occurred during the pre-transfusion phase in clinical services [17], as supported by the present study.

Also, the present study revealed that completing the blood sample label at the patient’s bedside and the blood and blood product request form by the doctor and the phlebotomist were among error-prone actions in the processes of phlebotomy and requesting blood and blood products from the department. Meanwhile, identifying patient, controlling the blood bags and blood products at the patient’s bedside, and checking the patient’s vital signs and recording in them in the transfusion monitoring form before transfusion, as the last safety measures before transfusion, require great care. This is one of the basic steps in improving the quality of clinical care, and failure to perform these steps correctly is the basis for subsequent errors in the hemovigilance process and the occurrence of blood transfusion reactions. As shown by the results of Karim’s study in Pakistan, accurate patient identification is crucial in blood transfusion. The most common errors leading to wrong blood transfusion are failure to identify patient and verify his/her identity correctly. Furthermore, human error is the only significant source of error in the patient identification. In addition, a blood sample with an incorrect label was identified as the main cause of blood bank’s distribution of incorrect blood or blood product [18]. Consistent with the current study, another study showed that most errors in the hemovigilance process occur during the completion and sending of request form for blood and blood products [19].

the findings of present study also showed that most errors that occur in the departments of Afzalipour Hospital, included “failure to obtain informed consent from the patient or his/her trusted companion for the transfusion” and “failure to provide full explanations to patient about the advantages and possible side effects of transfusion by the nurse and physician.“

In the study of Court et al. the physician and nurse’s failure to provide complete explanations about the advantages and possible side effects of transfusion and failure to obtain informed consent from the patient or his trusted companion were an indication of miscommunication between healthcare professionals and patients [20]. In addition, “absence of a witness at the patient’s bedside” and “absence of the nurse performing the transfusion at the patient’s bedside during the first 15 minutes of blood transfusion” were identified as the most common errors in the blood transfusion procedure. In a study by Najafpour et al., failure to monitor the patient’s signs and symptoms within the first 15 min of blood transfusion was identified as an error associated with a serious transfusion reaction. Since human factors play a crucial role in preventing complication related to blood transfusion, it is recommended that physicians and nurses receive training on blood transfusion instructions [21], which is consistent with the findings of present study.

According to the results of present study, incorrect crossmatching, using the slide test instead of tube test in determining the blood group, not using the standard crossmatch label after preparing the blood bags or blood products, not checking the blood bag or blood products and the receipt printout sent from the blood transfusion center by the blood bank personnel, using a non-standard cold box and not having a thermometer in the cold box during transportation accounted for the most errors. According to the findings of Elhence et al. the error in the standard crossmatch labels after the preparation of blood bag or blood product is one of the most common errors, and human error is the main factor in the majority of errors [22]. According to a study by Aalaei et al., an increase in RBC temperature during the blood transfer due to the use of a non-standard cold box between different departments may increase the RBC temperature outside the standard range, which is an error that can lead to RBC wastage [23].

According to the findings of present study, errors often occur in the clinical, human, equipment, support, and registration areas. Thus, in order to reduce these errors, perform safe blood and blood product transfusions, ensure the patient safety and prevent transfusion reactions, the following suggestions have been made by the experts and the research team: The physicians, nurses and blood bank personnel should receive a continuous education on the blood transfusion standards and instructions by the blood transfusion committee through educational workshops and practical training at the patient’s bedside. More supervision should be provided by the blood transfusion organization on the process of hospital’s hemovigilance system by increasing inspection programs, compiling educational pamphlets containing comprehensive information about the advantages and possible side effects of blood transfusion at the patient’s bedside, and incorporating a special educational unit of transfusion medicine into the curriculum of medical science programs [16, 19, 21].

Other solutions that appear to be effective in this regard include requiring the nurse or phlebotomist to send a copy of blood transfusion consent form to the blood bank at the same time as sending the blood sample and checking the recipient’s blood group for the second time using the slide test by another technician in the blood bank before sending the blood or product to the department, in order to prevent ABO incompatibilities and phlebotomy errors [24].

As equipment factors play a significant role in preventing blood transfusion side effects, the use of new identification systems for patient and blood products, such as Bloodloc, radio frequency identification tags for matching documents, blood group, and patient, and standard temperature monitoring device (STMD) will be an effective step in measuring and recording the temperature of blood bags and blood products during transportation [22, 25, 26].

## Limitation

Since this study was conducted by direct observation during the COVID-19 pandemic, it was not possible to enter sensitive departments, such as bone marrow transplant departments due to the spread of virus and infection. Consequently, the research team investigated other departments to examine the quality of hemovigilance process.

### Strengths

This study is one of the few studies in Iran that has identified errors in the hemovigilance process using the first two steps of Six Sigma model in a detailed manner based on different work shifts, labels, and sub-processes.

## Conclusion

The evaluation of hemovigilance process at Afzalipour Hospital revealed that the quality level of this process was low in that hospital. The results also showed that this process contains numerous errors, the majority of which occurring during sending a request from the blood bank to the blood transfusion organization and delivering the required bloods and blood products. We also found that most errors were happening at the night and evening shifts, mainly being related to clinical and registration labels. In this study, having completed only two steps of DMAIC cycle, we did not take any action or intervention to improve the hemovigilance process. However, by performing the initial steps of define and measure, all errors in the hemovigilance process were identified in a precise manner. In this regard, the sub-processes, work shifts and labels accounted for the highest number of errors. These findings can assist hospital managers and officials of healthcare industry to take necessary measures to reduce such errors in evening and night shifts in hospitals. This ultimately, would to prevent clinical and registration errors, particularly during the process of sending a request from the blood bank to the blood transfusion organization.

### Electronic supplementary material

Below is the link to the electronic supplementary material.

Supplementary Material 1

## Data Availability

The datasets analysed during the current study are available from the corresponding author on reasonable request.

## Abbreviations

SHOT: Serious Hazards of Transfusion
SIPOC: Suppliers, Inputs, Processes, Outputs, Customers
DMAIC: Define, Measure, Analyze, Improve, Control
HIS: Hospital Information System
DPMO: Defects Per Million Opportunities
DPU: Defects Per Unit
TRRF: Transfusion Reaction Reporting Form
STMD: Standard Temperature Monitoring Device

## References

[1] Lee SE, Morse BL, Kim NW (2022). Patient safety educational interventions: a systematic review with recommendations for nurse educators. Nurs open.

[2] Kakemam E, Gharaee H, Rajabi MR, Nadernejad M, Khakdel Z, Raeissi P (2021). Nurses’ perception of patient safety culture and its relationship with adverse events: a national questionnaire survey in Iran. BMC Nurs.

[3] Kumthekar AN, Sonune MS. Study of errors in pre analytical, analytical and post analytical phases of testing cycle at Central Clinical Laboratory of a tertiary hospital. Eur J Mol Clin Med.9(03):2022.

[4] Sahmoud S, Ashry EM, El Kalioby M, Kamel N (2021). Knowledge improvement of blood transfusion safety among pediatricians: post educational intervention. Transfus Med Rev.

[5] Muthuragavan S, Hariharan DA, Chitra DS (2021). Comparison of Cross Match and Transfusion ratio with utilization of Blood Components. Eur J Mol Clin Med.

[6] DEMİRAĞ H, Hintistan S (2020). Knowing and use situations of Hemovigilance System in the scope of blood transfusion safety of nurses: rural example. Bezmialem Sci.

[7] Cagliano AC, Grimaldi S, Rafele C (2021). A structured approach to analyse logistics risks in the blood transfusion process. J Healthc Risk Manage.

[8] Hamidi N, Ujang IRM, Awang S, Badaruddin NK, Hassan A, Mahmud SH (2022). Blood transfusion errors: where is the critical point?. J Health Manage.

[9] Nasir N, Shaikh U, Ali N, Hussain S (2020). Compliance of hand written transfusion requisition form and improvement after online request-a clinical audit. JPMA The Journal of the Pakistan Medical Association.

[10] Mora A, Ayala L, Bielza R, Ataúlfo González F, Villegas A (2019). Improving safety in blood transfusion using failure mode and effect analysis. Transfusion.

[11] McDermott O, Antony J, Bhat S, Jayaraman R, Rosa A, Marolla G (2022). Lean six Sigma in Healthcare: a systematic Literature Review on Challenges, Organisational Readiness and critical success factors. Processes.

[12] Elumalai V, Nachan FD, Ahamed S, Biswas S (2022). Rectification of defects occurring in the Gate Valve Manufacturing process by implementing six Sigma Methodology. ECS Trans.

[13] Ravichandran J (2017). A note on determination of sample size from the perspective of six Sigma Quality. J Mod Appl Stat Methods.

[14] Jindal A, Maini N. Six Sigma in blood transfusion services: A dream too big in a third world country? Vox Sanguinis. 2022. 10.1111/vox.13349 36102136

[15] Trusko B. In: Pexton C, Harrington H, Gupta P, editors. Improving healthcare quality and cost with six sigma. FT Press; 2007.

[16] Kaur G, Kaur G, Kaur P (2019). Nature and causes of errors in the blood transfusion chain–a step towards patient safety. ISBT Sci Ser.

[17] Sidhu M, Meenia R, Akhter N, Sawhney V, Irm Y (2016). Report on errors in pretransfusion testing from a tertiary care center: a step toward transfusion safety. Asian J Transfus Sci.

[18] Karim F, Moiz B, Shamsuddin N, Naz S, Khurshid M (2014). Root cause analysis of non-infectious transfusion complications and the lessons learnt. Transfus Apheres Sci.

[19] Maskens C, Downie H, Wendt A, Lima A, Merkley L, Lin Y (2014). Hospital-based transfusion error tracking from 2005 to 2010: identifying the key errors threatening patient transfusion safety. Transfusion.

[20] Court E, Robinson J, Hocken D (2011). Informed consent and patient understanding of blood transfusion. Transfus Med.

[21] Najafpour Z, Hasoumi M, Behzadi F, Mohamadi E, Jafary M, Saeedi M (2017). Preventing blood transfusion failures: FMEA, an effective assessment method. BMC Health Serv Res.

[22] Elhence P, Shenoy V, Verma A, Sachan D (2012). Error reporting in transfusion medicine at a tertiary care centre: a patient safety initiative. Clin Chem Lab Med (CCLM).

[23] Aalaei S, Amini S, Keramati MR, Shahraki H, Eslami S (2019). Monitoring of storage and transportation temperature conditions in red blood cell units: a cross-sectional study. Indian J Hematol Blood Transfus.

[24] Sindhulina C, Joseph N (2014). Addressing sample identification errors in a multispecialty tertiary care hospital in Bangalore. Vox Sang.

[25] Moiz B, Siddiqui AK, Sana N, Sadiq MW, Karim F, Ali N (2020). Documentation errors in transfusion chain: Challenges and interventions. Transfus Apheres Sci.

[26] Tiwari AK, Sharma P, Pandey PK, Rawat GS, Dixit S, Raina V (2015). A cost effective model for appropriate administration of red cell units and salvaging un-transfused red cell units by using temperature sensitive indicators for blood component transportation in a hospital setting. Asian J Transfus Sci.

