# Supplementary material for: Investigating the quality of hemovigilance process using the first two steps of Six Sigma model: a cross-sectional study
Source: BMC Health Serv Res. 2023 Oct 27;23:1169. doi: 10.1186/s12913-023-10113-6 (PMC10605775; doi:10.1186/s12913-023-10113-6)
Supplement: Supplementary file 1 — Supplementary Material 1 [file 12913_2023_10113_MOESM1_ESM.docx]

**S table 1- "Phlebotomy "**

| activities | label | initial actions | second-level actions | third-level actions | Observations (Yes or No) |
| --- | --- | --- | --- | --- | --- |
| pre-phlebotomy nursing actions | clinical | Checking physician's orders by a nurse |  |  |  |
|  | clinical | Patient identification by two nurses | Unconscious or emergency patient | Using an alias or file number |  |
|  | clinical |  | Conscious patient | Wristband |  |
|  | clinical |  | Conscious patient | Active identification (directly asking the patient for their full name, date of birth) |  |
|  | clinical | Informed consent from the patient/authorized companion (obtaining informed consent is mandatory at each stage of the patient's hospitalization |  |  |  |
| phlebotomy (in cases where the purpose is blood preparation, two CBC samples are taken except for pediatric, neonatal, and oncology departments( | clinical | Preparing the necessary phlebotomy equipment |  |  |  |
|  | clinical | Wearing gloves |  |  |  |
|  | clinical | Selecting an appropriate vein for phlebotomy |  |  |  |
|  | clinical | The tourniquet should not be tied tightly for a long time |  |  |  |
|  | clinical | The blood sample should be taken from a hand that is not receiving intravenous fluids or preferably from a site below the IV insertion site |  |  |  |
|  | clinical | If the blood sample is taken from the injection site, first discard 5 to 10 milliliters of blood and then collect the sample |  |  |  |
|  | clinical | Cleaning the blood sampling site with alcohol |  |  |  |
|  | clinical | Disposal of the needle tip in the safety box |  |  |  |
|  | clinical | Dispensing the blood sample into appropriate tubes using a needleless syringe (using an anticoagulant-containing tube and an EDTA-containing tube for CBC samples). Samples poured into anticoagulant-containing tubes must be gently mixed immediately by inverting 5-10 times (to perform wrist-crossing). A sample should be prepared in a plain/clot tube for blood group determination |  |  |  |
|  | clinical | If the patient is visiting the hospital for the first time, CBC and clot samples must be prepared for sending to the blood bank |  |  |  |
| Labeling blood sample on patient's bedside | registration | Patient's full name |  |  |  |
|  | registration | Admission code |  |  |  |
|  | registration | Department name |  |  |  |
|  | registration | Date of phlebotomy |  |  |  |
|  | registration | Time of sample collection |  |  |  |
|  | registration | Request code |  |  |  |
|  | registration | Name of phlebotomist |  |  |  |
|  | registration | Completing the label with legible handwriting |  |  |  |

**S table 2- "Requesting blood and blood products from the department"**

| activities | label | initial actions | second-level actions | third-level  actions | observations (Yes or No) |
| --- | --- | --- | --- | --- | --- |
| The diagnosis of the treating physician for the patient's need for blood or blood products is mandatory and a physician's order must include these items | registration | Type and quantity of blood units or blood products |  |  |  |
|  | registration | transfusion or reservation of blood |  |  |  |
|  | registration | Date of blood transfusion |  |  |  |
|  | registration | Time of blood transfusion |  |  |  |
|  | registration | If necessary, prescribing medication prior to blood transfusion to prevent reactions during transfusion |  |  |  |
| completion of the blood and blood product request form by the phlebotomist and the physician | registration | Patient information/profile | First and last name |  |  |
|  | registration |  | Father's name |  |  |
|  | registration |  | Date of birth |  |  |
|  | registration |  | ID number |  |  |
|  | registration |  | Request code |  |  |
|  | registration |  | Gender |  |  |
|  | registration |  | department |  |  |
|  | registration |  | Medical record number |  |  |
|  | registration | Medical History | History of transfusion in the past three months |  |  |
|  | registration |  | History of pregnancy in the past three months |  |  |
|  | registration |  | History of acute adverse reactions related to blood transfusion |  |  |
|  | registration |  | History of unexpected antibodies in the serum |  |  |
|  | registration | Do you need to prescribe medication prior to the illness? If yes | Name of medication |  |  |
|  | registration |  | Prescription method |  |  |
|  | registration | Reason for the need for blood transfusion or blood product | Diagnosis of the disease |  |  |
|  | registration |  | What is the reason for the need for blood or blood product, which of the following reasons applies |  |  |
|  | registration |  | If requesting red blood cell products, the hemoglobin level |  |  |
|  | registration |  | If requesting platelet products, the platelet count |  |  |
|  | registration |  | Patient's blood type and Rh factor (if known) |  |  |
|  | registration | Determination of the requested blood product types |  |  |  |
|  | registration | Date of need for blood and blood products |  |  |  |
|  | registration | Time of need for blood and blood products |  |  |  |
|  | registration | Recommended duration or rate of transfusion |  |  |  |
|  | registration | Treating physician | First and last name |  |  |
|  | registration |  | Signature |  |  |
|  | registration |  | Stamp |  |  |
|  | registration |  | Date |  |  |
|  | registration | Purpose of blood request | Blood reservation |  |  |
|  | registration |  | Preparation of blood for transfusion | Determination of blood group and crossmatch |  |
|  | registration | Information of the phlebotomist | Nurse or laboratory personnel |  |  |
|  | registration |  | First and last name |  |  |
|  | registration |  | time of phlebotomy |  |  |
|  | registration |  | Date of phlebotomy |  |  |
|  | registration |  | Signature of phlebotomist |  |  |
|  | registration |  | Stamp of phlebotomist |  |  |
|  | clinical |  | Control and supervision of obtaining the patient's informed consent by the phlebotomist |  |  |
| completion of the platelet apheresis request form by the requesting  physician | registration | Type of platelets |  |  |  |
|  | registration | Patient's first and last name |  |  |  |
|  | registration | Father's name |  |  |  |
|  | registration | Date of birth |  |  |  |
|  | registration | ID number |  |  |  |
|  | registration | Gender |  |  |  |
|  | registration | department |  |  |  |
|  | registration | Medical record number |  |  |  |
|  | registration | Date of request |  |  |  |
|  | registration | Desired date for blood product transfusion |  |  |  |
|  | registration | Illness |  |  |  |
|  | registration | Reason for the request |  |  |  |
|  | registration | Diagnosis of the disease |  |  |  |
|  | registration | Patient's platelet count |  |  |  |
|  | registration | Patient's blood type and Rh factor |  |  |  |
|  | registration | If performed, patient's HLA status |  |  |  |
|  | registration | Required platelet count (unit) |  |  |  |
|  | registration | Donors referred to the organization |  |  |  |
|  | registration | Inclusion of test sheet in case the donor's platelet count is mentioned |  |  |  |
|  | registration | Physician's name |  |  |  |
|  | registration | Physician's signature |  |  |  |
|  | registration | Medical board stamp |  |  |  |
|  | registration | Physician's phone number |  |  |  |
| completion of the irradiated blood and blood product request form | registration | Patient's first and last name |  |  |  |
|  | registration | Patient's blood type |  |  |  |
|  | registration | patient age |  |  |  |
|  | registration | Projected date for transfusion |  |  |  |
|  | registration | Provisional diagnosis |  |  |  |
|  | registration | Patient's hospital department |  |  |  |
|  | registration | Reason for the request of irradiated blood and blood product |  |  |  |
|  | registration | Treating physician's name |  |  |  |
|  | registration | Treating physician's stamp |  |  |  |
|  | support | Sending three copies of the irradiate form to the blood bank |  |  |  |
| Registration of request forms by nurses | registration | Registration of the request in the central HIS |  |  |  |
|  | support | Taking two printouts of each written request (the original and the second copy) |  |  |  |
| Delivery and sending of forms and blood samples by the hospital porters | support | Transfer of samples with covered containers under room temperature conditions by the patient |  |  |  |
|  | support | Gentle transportation of the samples |  |  |  |
|  | support | Delivery of both copies of the request forms (yellow and original) to the blood bank |  |  |  |

**S table 3- "Sending a request from the blood bank to the blood transfusion center**, **and delivering the required blood bags and blood products"**

| activities | label | initial actions | second-level actions | third-level actions | observations (Yes or No) |
| --- | --- | --- | --- | --- | --- |
| Completion of the blood and blood product request form by the blood bank officer | clinical | Checking the custom order point of stock |  |  |  |
|  | registration | Date of request |  |  |  |
|  | registration | Time of request |  |  |  |
|  | registration | First and last name of blood bank officer |  |  |  |
|  | registration | Signature of blood bank officer |  |  |  |
|  | registration | Determination of the type of blood product |  |  |  |
|  | registration | Number of blood products |  |  |  |
|  | registration | Blood bank inventory |  |  |  |
|  | support | For requesting customized radiology products, the blood bank needs to coordinate with the blood transfer station's distribution unit 15 to 20 minutes before the designated hours (10-11 am, 2-3 pm, and 10-11 pm) |  |  |  |
|  | support | For requesting customized washed blood products, the desired blood bag with the crossmatch is selected, then sent to the blood transfer organization, and coordination between the patient's physician and the blood transfer physician (via phone call) is necessary |  |  |  |
|  | support | If the organization accepts the request for washed blood products, the department informs the blood bank |  |  |  |
|  | support | After crossmatching fresh blood, the blood bank personnel send the product for washing to the organization |  |  |  |
| completion of the irradiated blood and blood product request form | registration | Name of blood bank officer |  |  |  |
|  | registration | blood group |  |  |  |
|  | registration | type of product |  |  |  |
|  | registration | Blood bank stamp |  |  |  |
|  | clinical | Determination of blood group if the requested blood is available in the blood bank |  |  |  |
| Completion of the platelet apheresis form | registration | Verification and signature of blood bank personnel |  |  |  |
|  | registration | Blood bank stamp |  |  |  |
|  | clinical | Determination of blood group |  |  |  |
| Delivery of necessary transportation equipment for the blood product by the blood bank personnel to the driver | equipment | cold box | Should have appropriate insulation |  |  |
|  | equipment |  | If it is being used multiple times, the box should be made of plastic or fiberglass and filled with high-density foam, washable, and free of sharp edges |  |  |
|  | equipment |  | The cold box should not be too large to easily lift it after placing the product in it |  |  |
|  | equipment |  | It should have sturdy handles |  |  |
|  | equipment |  | It should have a suitable width and height to easily accommodate the blood bag |  |  |
|  | equipment |  | Preferably, the box should have a unit identification number |  |  |
|  | equipment |  | A brief description of how to transport blood and blood products on the box should be provided |  |  |
|  | equipment |  | The box should have provisions for attaching transport documents (such as forms for the recipient, sender, contents, etc.) and a specific mark, such as an upward arrow, should be present on the box |  |  |
|  | equipment | Absorbent cloth |  |  |  |
|  | equipment | Plastic bag |  |  |  |
|  | equipment | Bubble wrap |  |  |  |
|  | equipment | Ice pack |  |  |  |
|  | equipment | Thermometer |  |  |  |
| Delivery of completed forms to the driver | support | The original and pink copy of the blood and blood product request form |  |  |  |
|  | support | The original and yellow copy of the platelet apheresis request form |  |  |  |
|  | support | irradiated blood and blood product request form |  |  |  |
| Driver's visit to the blood transfer organization at the scheduled time | support | The driver must have a special card (trained) |  |  |  |
|  | support | Delivery of the request form to the blood distribution personnel of the blood transfer organization |  |  |  |
|  | support | The driver's visit to the blood transfer organization at the appointed time |  |  |  |
|  | support | The driver's visit to the blood transfer organization with a special vehicle |  |  |  |
|  | support | Receiving the blood products by the driver from the blood distribution personnel |  |  |  |
| Transportation of RBC using a cold box by the driver | support | Placing an absorbent material such as paper on the bottom of the cold box |  |  |  |
|  | support | Placing the plastic bag inside the box |  |  |  |
|  | support | Before placing the bags in the cold box, the specifications of the blood bags should be matched with the product delivery form, which lists the products to be sent |  |  |  |
|  | support | After visual inspection, the bags should be placed inside the plastic bag inside the cold box |  |  |  |
|  | support | A temperature logger should be placed between the last two blood bags |  |  |  |
|  | support | An insulator (bubble wrap) should be placed over the blood bags |  |  |  |
|  | support | The insulator (bubble wrap) should be covered with an appropriate amount of ice pack, which has been frozen in a special freezer for preserving plasma |  |  |  |
|  | support | A copy of the form specifying the details of the sent blood units should be placed in a plastic cover and placed inside the cold box |  |  |  |
|  | support | The temperature should be recorded at the time of sending and receiving |  |  |  |
|  | support | Only one type of blood product should be packaged in the cold box |  |  |  |
|  | support | The temperature of the blood products should be maintained between 1 to 10 degrees Celsius |  |  |  |
|  | support | The maximum transportation time for red blood cells is one hour. (Please check the transportation time via Google Earth.) |  |  |  |
| Transportation of plasma using a cold box by the driver | support | An absorbent material such as paper should be placed on the bottom of the cold box |  |  |  |
|  | support | The plastic bag should be placed inside the box |  |  |  |
|  | support | Before placing the bags in the cold box, the specifications of the plasma bags should be matched with the product delivery form, which lists the products to be sent |  |  |  |
|  | support | After visual inspection, the bags should be placed inside the plastic bag inside the cold box |  |  |  |
|  | support | A temperature logger should be placed between the last two plasma bags |  |  |  |
|  | support | Sufficient ice boxes should be placed directly on the plasma bags. The ice boxes for transporting plasma should have been stored in a freezer at a temperature within the range of plasma storage temperature or cold |  |  |  |
|  | support | A copy of the form specifying the details of the sent blood units should be placed in a plastic cover and placed inside the cold box bag. The plastic bag should be sealed and covered with bubble wrap |  |  |  |
| Transportation of plasma using a cold box by the driver | support | The appropriate temperature for transporting platelets is between 20 to 24 degrees Celsius |  |  |  |
|  | support | For transporting platelets, the platelet agitator should be removed from the incubator immediately before transportation and then placed in plastic bags (preferably bubble wrap) |  |  |  |
|  | support | The specialized box for transportation at room temperature is placed and, for each size that fits within the box, platelets can be placed inside it |  |  |  |
|  | clinical | As soon as platelets arrive at their destination, they should be placed inside the shaker |  |  |  |
|  | support | A copy of the form specifying the details of the sent blood units should be placed in a plastic cover and placed inside the cold box. The plastic bag should be sealed and covered with bubble wrap |  |  |  |
| Completion of the blood and blood product request form by the hospital representative for receiving the product from the blood transfer station | registration | The name and surname of the recipient of the blood product from the blood transfusion center |  |  |  |
|  | registration | The signature of the recipient of the blood product from the blood transfusion center |  |  |  |
|  | registration | The date of receiving the blood product from the blood transfusion center |  |  |  |
|  | registration | The time of receiving the blood product from the blood transfusion center |  |  |  |
|  | registration | Receiving a printed receipt for distribution of the blood product |  |  |  |
|  | registration | Receiving a printed receipt for distribution of the blood product | Sending number |  |  |
|  | registration |  | Letter number |  |  |
|  | registration |  | Sender unit |  |  |
|  | registration |  | Receiving unit |  |  |
|  | registration |  | Transportation means |  |  |
|  | registration |  | Sender postal address |  |  |
|  | registration |  | Receiving postal address |  |  |
|  | registration |  | Sending date |  |  |
|  | registration |  | Sending time |  |  |
|  | registration |  | Sender's full name |  |  |
|  | registration |  | Sender's signature |  |  |
|  | registration |  | Receiving date |  |  |
|  | registration |  | Receiving time |  |  |
|  | registration |  | Recipient's full name |  |  |
|  | registration |  | Recipient's signature |  |  |
|  | registration |  | The list of sent products according to the request received from the blood bank | Expiration date |  |
|  | registration |  |  | blood group |  |
|  | registration |  |  | Type of blood product |  |
|  | registration |  |  | Donation number |  |
|  | registration |  | Transportation information | Type of transportation means |  |
|  | registration |  |  | Number of gelatin bags |  |
|  | registration |  |  | Compliance of transportation requirements with valid certification documents |  |
|  | registration |  |  | Listing of non-compliance issues with transportation requirements |  |
|  | registration |  | The action taken in case of non-compliance | Cold box ambient temperature |  |
|  | registration |  |  | Name and code of temperature device provider |  |
|  | registration |  |  | Serial number of the security label |  |
|  | registration |  |  | Matching the security label barcode on the cold box with the sending form |  |
|  | registration |  |  | Integrity of the security label |  |
|  | registration |  |  | Number of bags |  |
|  | registration |  |  | Matching the number of bags with the sending form |  |
|  | support | The original copy is given by the driver to the blood department |  |  |  |
|  | support | The second copy, along with the cold box containing the blood product, is returned to the blood bank by the driver and is archived in the blood bank |  |  |  |
| completion of the irradiated blood and blood product request form by Blood transfusion center | registration | Delivery date in blood distribution |  |  |  |
|  | registration | Delivery date in blood distribution |  |  |  |
|  | registration | The name and surname of the blood distribution specialist |  |  |  |
|  | registration | The signature of the blood distribution specialist |  |  |  |
|  | registration | blood transfer center |  |  |  |
|  | support | The relevant copy remains in the blood department |  |  |  |
| Completion of the platelet apheresis request form by the blood transfusion center | registration | Estimated platelet count per bag |  |  |  |
|  | registration | Blood product delivery date |  |  |  |
|  | registration | Product delivery time |  |  |  |
|  | registration | Signature of Apheresis Platelet Officer |  |  |  |
|  | registration | Signature of Apheresis Platelet Medical Officer |  |  |  |
|  | support | Both copies will be returned to the blood bank by the driver |  |  |  |
| Completion of the platelet apheresis request form by the blood transfusion center | clinical | Bags should not have bubbles |  |  |  |
|  | clinical | Bags should have labels |  |  |  |
|  | clinical | Bags should not be contaminated |  |  |  |
|  | clinical | Check the temperature on the thermometer appropriate for the type of blood product |  |  |  |
|  | clinical | Match the blood products with the request |  |  |  |
|  | registration | Check and complete the printed distribution receipt | Type of transportation means |  |  |
|  | registration |  | Number of gelatin bags |  |  |
|  | registration |  | Compliance of transportation requirements with valid certification documents |  |  |
|  | registration |  | Listing of non-compliance issues with transportation requirements |  |  |
|  | registration |  | Actions taken in case of non-compliance |  |  |
|  | registration |  | Cold box ambient temperature |  |  |
|  | registration |  | Name and code of temperature device provider |  |  |
|  | registration |  | Serial number of the security label |  |  |
|  | registration |  | Matching the security label barcode on the cold box with the sending form |  |  |
|  | registration |  | Integrity of the security label |  |  |
|  | registration |  | Number of bags |  |  |
|  | registration |  | Matching the number of bags with the sending form |  |  |
|  | registration |  | Names of the two approvers |  |  |
|  | registration |  | Signatures of the two approvers |  |  |
|  | registration | Registration of the recipient driver |  |  |  |
|  | registration | Name of the recipient of the blood product at the blood bank |  |  |  |
|  | registration | Signature of the recipient of the blood product at the blood bank |  |  |  |
|  | registration | Recording the date of delivery of the blood product at the blood bank |  |  |  |
|  | registration | Recording the time of delivery of the blood product at the blood bank |  |  |  |
|  | clinical | Determining the blood group of each received pack cell by the blood bank personnel to ensure the accuracy of the designated blood group |  |  |  |
| If there are any issues with the received blood products | registration | Completion of the form to register the returned blood and blood products by the blood bank personnel | Name of the department |  |  |
|  | registration |  | Return registration date |  |  |
|  | registration |  | Return time |  |  |
|  | registration |  | Donation number |  |  |
|  | registration |  | Type of blood product |  |  |
|  | registration |  | blood group |  |  |
|  | registration |  | RH |  |  |
|  | registration |  | Hospital name |  |  |
|  | registration |  | Reason for return |  |  |
|  | registration |  | Sender's name |  |  |
|  | registration |  | Sender's signature |  |  |
| Placing in the refrigerator based on the principle that the product with the earliest expiration date is put first | clinical |  |  |  |  |

**S table 4- "Application preparation by the blood bank and sending it to the department"**

| activities | label | initial actions | second-level actions | observations (Yes or No) |
| --- | --- | --- | --- | --- |
| Review of the request received from the department by the blood bank personnel | clinical | Matching hospital porter information on the sample with patient information on the request form |  |  |
|  | clinical | Checking the seal and signature of the physician and nurse on the request form |  |  |
|  | registration | Entering patient code into the HIS |  |  |
|  | clinical | Matching the requested blood product in the system with the request form |  |  |
|  | clinical | Archiving the second copy of the blood and blood product request form |  |  |
|  | clinical | In case of inconsistency between the information in HIS and the written request, notifying the department by phone to correct the request |  |  |
|  | clinical | If the patient information on the label is unreadable or incomplete, or if the label lacks the seal and signature of the physician and nurse on the request form, return the test tube and request form to the department officials |  |  |
|  | clinical | In case of blood reservation | Storage of blood samples in the blood bank for 3 days |  |
|  | clinical |  | Checking the stock for having reserved blood type |  |
|  | clinical | In case of blood reservation | Determination of blood group |  |
|  | clinical |  | crossmatch |  |
| Determination of blood group | clinical | Using the tube method |  |  |
|  | clinical | cell type test | The blood bank personnel transfer at least one milliliter of whole blood through a sampler to a ten-milliliter plastic tube |  |
|  | clinical |  | add saline to the red blood cells (2-3 times) |  |
|  | clinical |  | They centrifuge it for 1 to 3 minutes at 1000 G or 2500 RPM with a tabletop centrifuge and each time remove excess antibody by pouring off the tube (2-3 times) |  |
|  | clinical |  | They transfer 300 microliters of washed packed red blood cells to a tube containing 7.9 milliliters of 0.9% saline through a sampler |  |
|  | clinical |  | They cover the tube with prefill and mix the red blood cells thoroughly with 3 to 5 percent saline solution by gently inverting the tube several times to make a suspension |  |
|  | clinical |  | They add anti-A, anti-B, and anti-D blood group antibodies through a sampler to three clean 75*12 mm tubes. The amount of antibodies should be equal to the 3-5 percent suspension |  |
|  | clinical |  | They add the red blood cell suspension (2-5 percent) to each tube through a sampler |  |
|  | clinical |  | They gently mix the contents of the tubes and centrifuge the tubes for 15 to 30 seconds at 900 G to 1000 rpm or 2500 rpm with a calibrated centrifuge according to the manufacturer's instructions |  |
|  | clinical |  | They gently shake the tubes to disperse the red cell mass into a free suspension. They observe for any agglutination or hemolysis using a concave mirror |  |
|  | clinical |  | They grade and interpret the reaction results |  |
|  | registration |  | They record the reaction results immediately in the laboratory notebook |  |
|  | clinical | back type test | This test is performed in clot tubes, not in CBC tubes |  |
|  | clinical |  | B cells are made from the blood of a patient with B positive blood group, A cells are made from the blood of a patient with A positive blood group, and O cells are made from the blood of a patient with O positive blood group |  |
|  | clinical |  | To make cells, whole blood is centrifuged three times in 1 to 3 minutes at 900 to 1000 G or 2500 RPM and the excess antigen is removed, and then a suspension is prepared by adding saline solution |  |
|  | clinical |  | Then, the patient's blood sample is centrifuged to separate the serum from the red blood cells |  |
|  | clinical |  | They examine the serum or plasma inside the tubes for the presence of hemolysis |  |
|  | clinical |  | They add 2 to 3 drops of the patient's serum to two clean 12 x 75 ml plastic tubes labeled as A1, B, and O |  |
|  | clinical |  | A, B, O suspensions are added to their respective tubes with a sampler |  |
|  | clinical |  | The contents inside the tubes are mixed gently and centrifuged with a calibrated centrifuge for 15 to 30 seconds at 900 to 1000 G or 2500 RPM. |  |
|  | clinical |  | They gently shake the tube to disperse the red cell mass into a free suspension and observe and examine for any agglutination using a concave mirror |  |
|  | clinical |  | They grade and interpret the reaction results |  |
|  | registration |  | record the reaction results immediately in the laboratory notebook |  |
|  | registration |  | Record the result of blood group determination in the HIS |  |
|  | clinical |  | Matching the results of cell type and back type tests |  |
|  | clinical |  | Cell type and back type tests should preferably be done by two people separately |  |
|  | clinical |  | Before every blood transfusion, cell type and back type test should be done |  |
| crossmatch | clinical | The time frame for performing blood typing and crossmatching tests is one hour |  |  |
|  | clinical | Crossmatching should be performed for each unit of blood, even if the blood group is known |  |  |
|  | clinical | Blood bank personnel label the tubes that are being tested with the blood sample of each donor and the patient's serum |  |  |
|  | clinical | They prepare a three percent (3%) red blood cell (RBC) suspension from the donor's blood |  |  |
|  | clinical | They add 2 drops of patient serum or plasma to each of the tubes |  |  |
|  | clinical | They add 1 drop of the donor's red blood cell suspension) 2-5%) to each of the corresponding tubes |  |  |
|  | clinical | They mix the contents of the tubes and then centrifuge them in a calibrated serological centrifuge for 30 to 15 seconds at a speed of 900 to 1000 G or 2500 RPM |  |  |
|  | clinical | They gently agitate the tubes to free any clumped cells and check for the presence of any agglutination |  |  |
|  | clinical | They read, interpret, and immediately record the test results. If the agglutination is resolved, it is considered compatible, and the process continues. However, if the agglutination persists, the patient may have cold agglutinin and the cross-matching process may not necessarily be stopped. If subsequent steps are negative, the blood can be made compatible by warming it according to the protocol |  |  |
|  | clinical | They add two drops of 22% albumin solution to the tube |  |  |
|  | clinical | They incubate the tube containing 22% albumin solution for 30 minutes at 37 degrees Celsius (in a water bath or incubator such as a BUNN-MARIE) |  |  |
|  | clinical | After the specified time, they centrifuge the tube in a calibrated serological centrifuge for 15 to 30 seconds at a speed of 900 to 1000 G or 2500 RPM |  |  |
|  | clinical | Blood bank personnel gently agitate the tubes to free any clumped cells and check for the presence of any agglutination. |  |  |
|  | clinical | They examine the reaction results and grade them |  |  |
|  | registration | They immediately record the reaction results in the laboratory record book |  |  |
|  | clinical | Then, they wash the contents of the tube three to four times with 0.9% saline solution and, in the final step, they discard the saline solution |  |  |
|  | clinical | They add anti-human globulin to the tube according to the manufacturer's instructions |  |  |
|  | clinical | They mix the contents of the tube and centrifuge it in a calibrated serological centrifuge for 15 to 30 seconds at a speed of 900 to 1000 G or 2500 RPM |  |  |
|  | clinical | They gently agitate the tube to free any clumped cells and check for the presence of any agglutination. They first examine the tube macroscopically and in cases of doubt, a microscopic examination is recommended |  |  |
|  | clinical | They examine the reaction results and grade them |  |  |
|  | clinical | If a negative reaction is observed, they add a drop of sensitized red blood cells to the tube containing anti-human globulin |  |  |
|  | clinical | They mix the contents of the tube and centrifuge it in a calibrated serological centrifuge for 15 to 30 seconds at a speed of 900 to 1000 G or 2500 RPM |  |  |
|  | clinical | They gently agitate the tubes to free any clumped cells and check for the presence of any agglutination |  |  |
|  | clinical | They immediately record the reaction results in the laboratory record book |  |  |
|  | clinical | If all the bags are incompatible with the patient's blood, additional tests will be performed to investigate the root cause of the issue for the patient. If the blood bank staff cannot find a solution, they will seek assistance from the personnel responsible for blood transfusion in the organization |  |  |
| Preparation of cryo and preparation of FFP (fresh frozen plasma) | clinical | Blood bank personnel remove the frozen product from the freezer and record the start time of the thawing process in the appropriate record book |  |  |
|  | clinical | Using a BUNN-MARIE for thawing the product, they first check and record the temperature of the BUNN-MARIE. Then, they place the frozen product inside an insulated bag (or appropriate compartment) and put it in the BUNN-MARIE |  |  |
|  | clinical | If any tears or leaks are observed in the product bag, blood bank personnel record the bag number and the reason for disposal in the waste record book. Then, they dispose of the product bag along with the insulated bag according to the hospital's internal guidelines |  |  |
|  | registration | To write "melting point" on FFP and Cryo |  |  |
|  | registration | To write "melting date" on FFP and Cryo |  |  |
|  | clinical | Cryo must be used within 6 hours of melting at ambient temperature |  |  |
|  | clinical | If not used after melting, FFP can be used up to 24 hours at a temperature range of 1 to 6 degrees Celsius |  |  |
| Preparation of platelets | support | Sending a platelet request to the blood transfer organization |  |  |
|  | clinical | Maintaining in a shaker incubator at a temperature of 20 to 25 degrees Celsius in the blood bank until the hospital porter comes to receive it |  |  |
| Preparation of apheresis platelets | registration | Confirmation and signature of blood bank personnel on the platelet apheresis request form |  |  |
|  | registration | The stamp of the blood bank on the platelet apheresis request form |  |  |
|  | clinical | Determination of blood group |  |  |
|  | support | The platelet apheresis request is sent from the blood bank to the blood transfer organization by a driver |  |  |
|  | clinical | Maintaining in a shaker incubator at a temperature of 20 to 24 degrees Celsius in the blood bank until the patient comes to receive it |  |  |
| Preparation of irradiated blood or blood product | registration | Completing the form for requesting irradiated blood or blood product | The name of the blood bank manager |  |
|  | registration |  | The blood bank stamp |  |
|  | registration |  | If the requested blood is available in the blood bank and a crossmatch has been performed on it, the crossmatched donation number should be written on it |  |
|  | support |  | Sending a request of irradiated blood or blood product |  |
|  | registration |  | type of blood product |  |
|  | registration |  | blood group |  |
|  | registration |  | Donation number |  |
| Preparation of blood bag or blood product by blood bank personnel | clinical | Applying the standard cross-match label on the blood or blood product |  |  |
|  | registration | Writing patient information and blood or blood product details on the label | Patient's name |  |
|  | registration |  | blood group and patient RH |  |
|  | registration |  | department |  |
|  | registration |  | ID number |  |
|  | registration |  | product name |  |
|  | registration |  | Bag label number |  |
|  | registration |  | Blood bag RH group |  |
|  | registration |  | Expiration date |  |
|  | registration |  | Crossmatch date |  |
|  | registration |  | Storage conditions |  |
|  | registration | Writing legible patient information and blood or blood product details on the label |  |  |
| Contacting the department via phone to prepare the blood product and check the mentioned items in the request | support | Department name |  |  |
|  | support | Type of blood product |  |  |
|  | support | Patient information |  |  |
| Completing the form for the specifications of the blood product sent from the blood bank (FFP, cryo, and platelets) | registration | Patient's first and last name |  |  |
|  | registration | Father's name |  |  |
|  | registration | department |  |  |
|  | registration | Patient file number |  |  |
|  | registration | ID number |  |  |
|  | registration | Blood group and patient RH |  |  |
|  | registration | date of birth |  |  |
|  | registration | gender |  |  |
|  | registration | Date of blood or blood product transfusion need |  |  |
|  | registration | time of blood or blood product transfusion need |  |  |
|  | registration | The name of the requested blood product by the physician and the quantity |  |  |
|  | registration | The time of sending the blood product |  |  |
|  | registration | The date of sending the blood product |  |  |
|  | registration | The first and last name of the sender |  |  |
|  | registration | The name of the recipient |  |  |
|  | registration | The signature of the sender |  |  |
|  | registration | The name of the blood product |  |  |
|  | registration | The bag number |  |  |
|  | registration | The expiration date |  |  |
|  | registration | The blood type and Rh factor of the sent blood product |  |  |
|  | registration | It is completed in three copies |  |  |
|  | support | The yellow copy is archived in the blood bank |  |  |
| Completing the form for the specifications of the blood bag and blood product sent from the blood bank | registration | The name of the blood product |  |  |
|  | registration | The expiration date of the blood product |  |  |
|  | registration | The blood bag number |  |  |
|  | registration | The blood type and Rh factor of the sent blood product from the blood bank |  |  |
|  | registration | Antibody screening |  |  |
|  | registration | Blood group and patient RH |  |  |
|  | registration | Cross match |  |  |
|  | registration | The date of the test |  |  |
|  | registration | department |  |  |
|  | registration | Patient's first and last name |  |  |
|  | registration | Father's name |  |  |
|  | registration | ID number |  |  |
|  | registration | Patient file number |  |  |
|  | registration | Date of birth |  |  |
|  | registration | Date of blood or blood product transfusion need |  |  |
|  | registration | Time of blood or blood product transfusion need |  |  |
|  | registration | The name of the requested blood product by the physician |  |  |
|  | registration | The first and last name of the person who performed the test |  |  |
|  | registration | The date of sending the blood product |  |  |
|  | registration | The time of sending the blood product |  |  |
|  | registration | The first and last name of the sender |  |  |
|  | registration | The name of the recipient |  |  |
|  | registration | The signature of the sender |  |  |
|  | registration | It is completed in three copies |  |  |
|  | support | The yellow copy is archived in the blood bank |  |  |
|  | registration | This form must be completed for each bag of blood |  |  |
|  | clinical | From the time the blood product is taken out of the blood bank refrigerator until the start of the injection to the patient, it should not take more than 30 minutes |  |  |
| Handing over the blood or blood product to the hospital porter | support | Sending a hospital porter to the blood bank by the department along with a cold box |  |  |
|  | support | Delivery of blood bags to a patient's department one by one with a time interval of 1 to 4 hours, if necessary, the department will divide them into multiple packs |  |  |
|  | support | Delivery of FFP, platelets, and cryo, maximum of 5 products per form |  |  |
|  | support | The forms are given to the hospital porter along with the blood products | The original and pink version of the specification form for blood bags and products sent from the blood bank |  |
|  | support |  | The original and pink copy of the product specification form sent from the blood bank |  |
|  | support |  | The original version of the request form for blood and blood products |  |
|  | support |  | irradiated blood or blood product request form |  |
|  | support |  | The original copy of the platelet apheresis request form |  |
|  | support | The hospital porter places the blood products in the cold box |  |  |
|  | support | The hospital porter delivers the cold box containing the blood products along with the relevant forms to the department |  |  |
| Registering hospital porter information in the dedicated book by blood bank personnel | registration | date |  |  |
|  | registration | Patient's first and last name |  |  |
|  | registration | department |  |  |
|  | registration | The time of sample arrival |  |  |
|  | registration | Blood group and patient RH |  |  |
|  | registration | The type of blood product |  |  |
|  | registration | The expiration date of the blood bag |  |  |
|  | registration | The bag number |  |  |
|  | registration | Blood group and patient RH |  |  |
|  | registration | The crossmatch result |  |  |
|  | registration | The person in charge of the compatibility test |  |  |
|  | registration | The time of delivering the blood product to the department |  |  |
|  | registration | The date of delivering the blood product to the department |  |  |
|  | registration | The name of the recipient |  |  |
|  | registration | The signature of the recipient |  |  |
|  | registration | Final status (consumption or return) |  |  |
| Proper storage of blood and blood products in the department by personnel until the time of injection (storage location, whether shaking is required or not, etc). | clinical | Proper storage of blood and blood products in the department by the staff until the time of transfusion (storage location, whether shaking is required or not, etc). |  |  |

**S table 5- "Transfusing blood and blood products in the department"**

| activities | label | initial actions | second-level  actions | Third-level  actions | fourth-level actions | fifth-level actions | observations (Yes or No) |
| --- | --- | --- | --- | --- | --- | --- | --- |
| Checking the physician's order by two nurses (injector and witness) | clinical | The nurse responsible for administering the blood or blood product |  |  |  |  |  |
|  | clinical | Blood or blood product transfusion by one nurse |  |  |  |  |  |
|  | clinical | Witness presence is mandatory (the witness must be a supervisor) |  |  |  |  |  |
| Completing the designated sections of the blood transfusion monitoring form or red blood cell product monitoring form prior to transfusion, or completing the fresh frozen plasma, platelet, or cryo monitoring forms prior to transfusion | clinical | Patient information and status of sent products | Patient identification can be done through questioning the patient (if conscious), checking the patient's wristband, and using the medical record number |  |  |  |  |
|  | clinical |  | Matching the patient's identity with the information on the blood request form and the blood product specification form |  |  |  |  |
|  | clinical |  | Matching the blood group and donor number information on the blood bag or blood product with the information on the blood product specification form |  |  |  |  |
|  | clinical |  | The expiration date of the blood product |  |  |  |  |
|  | clinical |  | Checking the physical appearance of the blood bag | Leakage |  |  |  |
|  | clinical |  |  | Unnatural color |  |  |  |
|  | clinical |  |  | hemolysis |  |  |  |
|  | clinical |  |  | the presence of a clot |  |  |  |
|  | clinical |  |  | The presence of turbidity |  |  |  |
|  | clinical |  |  | The presence of gas in the bag |  |  |  |
|  | clinical |  |  | inflated bag |  |  |  |
|  | clinical |  |  | Density |  |  |  |
|  | clinical |  |  | Unhealthy label |  |  |  |
|  | registration |  | The name of the person administering the transfusion |  |  |  |  |
|  | registration |  | The signature of the person administering the transfusion |  |  |  |  |
|  | registration |  | The name of the witness |  |  |  |  |
|  | registration |  | The signature of the witness |  |  |  |  |
|  | clinical |  | If any of the above items cannot be confirmed or verified | Blood transfusion is not performed |  |  |  |
|  | support |  |  | Returning the blood bag to the blood bank |  |  |  |
|  | clinical |  |  | Report to hemovigilance physician or blood transfusion consultant doctor |  |  |  |
|  | registration | Record the delivery time of the blood product and how to transfusion it | The time of delivery of the blood bag to the department |  |  |  |  |
|  | registration |  | The date of delivery of the blood bag to the department |  |  |  |  |
|  | registration |  | The size or color of the needle used |  |  |  |  |
| If any of the products are not used, they must be returned to their previous conditions | clinical | If the RBC product is not used, it should be promptly returned to the blood bank. This product can be stored for a maximum of 30 minutes at 20-24 degrees Celsius after leaving the blood bank in the storage area |  |  |  |  |  |
|  | clinical | After thawing, cryo can only be stored and used at room temperature for a maximum of 6 hours |  |  |  |  |  |
|  | clinical | If not used after thawing, thawed FFP can be refrigerated at 1-6 degrees Celsius and can still be used as fresh frozen plasma for up to 24 hours |  |  |  |  |  |
| Patient preparation by the nurse prior to blood transfusion | clinical | The physician provides a detailed explanation to the patient about the benefits and potential risks of blood transfusion, and also informs the patient that if they experience any adverse reactions during the transfusion, they should notify the nurse immediately. The nurse then provides additional information to the patient as needed |  |  |  |  |  |
|  | clinical | Checking the informed consent form from the patient regarding blood transfusion |  |  |  |  |  |
| Preparation of the nurse prior to blood transfusion | clinical | Hand disinfection |  |  |  |  |  |
|  | clinical | Checking the patient's vital signs (temperature, blood pressure, pulse rate, respiratory rate, general condition) and recording them in the monitoring form prior to transfusion |  |  |  |  |  |
|  | clinical | Checking the patient's skin (itchiness, rash, etc.) |  |  |  |  |  |
| Two nurses, the witness and the injector, transfusion the blood or blood product into the patient at the bedside | clinical | If necessary, the nurse dilutes the blood according to the physician's discretion using a Y-set, which has one end connected to an air vent and the other end connected to a bag of normal saline and another end connected to the blood bag, and then administers the blood transfusion |  |  |  |  |  |
|  | clinical | During the first 15 minutes of the transfusion, it should be administered very slowly (the infusion rate is 2 milliliters or 30 drops per minute) |  |  |  |  |  |
|  | clinical | During this 15-minute period, the nurse must be present at the patient's bedside and monitor them closely |  |  |  |  |  |
|  | clinical | If no adverse reactions are observed within 15 minutes of starting the transfusion, the nurse adjusts the flow rate according to the physician's orders |  |  |  |  |  |
|  | clinical | Checking the patient's vital signs (temperature, blood pressure, pulse rate, respiratory rate, general condition) in the first quarter, half an hour later, and every hour thereafter, and recording them in the monitoring form for blood transfusion |  |  |  |  |  |
|  | clinical | Checking the patient's vital signs (temperature, blood pressure, pulse rate, respiratory rate, general condition) during the first, second, third, fourth, and fifth product transfusion, and recording them in the monitoring form for product transfusion |  |  |  |  |  |
|  | clinical | If the patient shows signs and symptoms of a reaction to the blood or blood product, the blood or blood product infusion should be discontinued and the physician should be notified immediately |  |  |  |  |  |
|  | clinical | If there is a change in the patient's vital signs, it is necessary to discontinue the blood transfusion | A temperature of 38 degrees Celsius |  |  |  |  |
|  | clinical |  | A blood pressure increase or decrease of 3 degrees |  |  |  |  |
|  | clinical |  | A pulse rate of more than 20 beats |  |  |  |  |
|  | clinical |  | A respiratory rate increase of 8 or more breaths per minute, or a respiratory rate of more than 28 breaths per minute |  |  |  |  |
|  | clinical |  | A decrease in blood oxygen level |  |  |  |  |
|  | clinical |  | Poor general condition of the patient |  |  |  |  |
|  | clinical |  | Abnormal urine appearance (in the case of having a catheter, check for blood and volume) |  |  |  |  |
|  | clinical | The normal saline infusion should be established (in case of TRALI reaction, normal saline should be administered, but in case of TACO reaction, normal saline should not be administered) |  |  |  |  |  |
|  | clinical | The patient's vital signs (temperature, blood pressure, pulse rate, respiratory rate, general condition) should be checked |  |  |  |  |  |
|  | clinical | The patient's identity, blood bag, and medical records are reviewed again, and the physician is informed |  |  |  |  |  |
|  | clinical | If a discrepancy is observed | Do not continue any transfusion under any circumstances |  |  |  |  |
|  | clinical |  | The priority order for contacting is as follows: Resident of the third year (if not available, then resident of the second and first years respectively) |  |  |  |  |
|  | clinical | If no discrepancy is observed | Severe symptoms | The transfusion should not continue under any circumstances |  |  |  |
|  | clinical |  |  | Contacting the third-year resident (if not available, contact the second-year and first-year residents in that order) takes priority |  |  |  |
|  | clinical |  | Mild symptoms | The blood transfusion is first discontinued. Then, with the physician's approval, the injection is slowly resumed, and the nurse checks the patient's bedside for at least 15 minutes after the onset of symptoms | Deterioration of symptoms | The transfusion should not continue under any circumstances |  |
|  | clinical |  |  |  |  | Continue the transfusion |  |
|  | clinical |  |  |  | Stable condition of the patient |  |  |
|  | clinical | It is better to administer each unit of blood for at least 2 hours, and the maximum infusion time is 4 hours |  |  |  |  |  |
|  | clinical | The duration of platelet transfusion is 30 minutes, and the maximum infusion time is 4 hours |  |  |  |  |  |
|  | clinical | The duration of plasma transfusion takes around 30 to 120 minutes (maximum 4 hours) to complete |  |  |  |  |  |
|  | clinical | The duration of cryoprecipitate transfusion takes around 10 to 30 minutes to complete |  |  |  |  |  |
|  | clinical | If the patient has received the complete blood or blood product, the empty bag should be disposed of in the yellow bin |  |  |  |  |  |
|  | clinical | If, for any reason, the blood or blood product is not completely administered, it should be returned to the blood bank for disposal |  |  |  |  |  |
| When the blood transfusion is completed or if it is not performed for any reason, the nurse completes the following sections in the monitoring form | registration | date of blood transfusion |  |  |  |  |  |
|  | registration | time of blood transfusion |  |  |  |  |  |
|  | registration | End time of the infusion |  |  |  |  |  |
|  | registration | The volume of the transfused product |  |  |  |  |  |
|  | registration | Whether a transfusion reaction occurred or not |  |  |  |  |  |
|  | registration | If the product was not transfused or returned to the blood bank, the reason should be documented as well |  |  |  |  |  |
| If an adverse reaction occurs, the potential adverse reaction report form should be completed by: | registration | nurse | patient information |  |  |  |  |
|  | registration |  | Clinical status |  |  |  |  |
|  | registration |  | injectable products |  |  |  |  |
|  | registration |  | Signs, clinical symptoms, and laboratory results |  |  |  |  |
|  | registration | physician | Diagnosis and severity of a condition |  |  |  |  |
|  | registration |  | The therapeutic interventions performed after the occurrence of an adverse reaction due to blood or product transfusion |  |  |  |  |
|  | registration |  | The causality of an adverse event |  |  |  |  |
|  | registration |  | The clinical condition of the patient related to the reaction caused by the transfusion of blood or blood product |  |  |  |  |
|  | registration | Related personnel | The name and surname of the injecting nurse |  |  |  |  |
|  | registration |  | Seal of the injecting nurse |  |  |  |  |
|  | registration |  | First and last name of the attending physician |  |  |  |  |
|  | registration |  | signature of the attending physician |  |  |  |  |
|  | registration |  | Seal of the attending physician |  |  |  |  |
|  | registration |  | date |  |  |  |  |
|  | registration |  | First and last name of the senior hemovigilance physician |  |  |  |  |
|  | registration |  | The signature of a hemovigilance specialist |  |  |  |  |
|  | registration |  | The seal of a hemovigilance specialist |  |  |  |  |
|  | registration |  | date |  |  |  |  |
|  | registration | The white copy of the monitoring form is attached to the patient's file, while the pink copy of the monitoring form, along with the adverse reaction form, CBC and UA blood samples, blood bag, and serum set, are sent by the patient to the blood bank and laboratory for further investigation |  |  |  |  |  |
|  | registration | If a severe adverse reaction such as TRALI occurs, it is necessary to take a chest X-ray |  |  |  |  |  |
|  | registration | If there is an increase in temperature of more than 1.5 degrees Celsius after blood transfusion, the blood bag and patient's blood sample should be returned to the blood transfusion organization for culturing (in addition to being checked at the blood bank) |  |  |  |  |  |
|  | registration | A new blood sample is sent from the department to the blood bank for re-determination of the blood type and crossmatching |  |  |  |  |  |
|  | registration | If the blood or blood product is not connected, the white and pink copies of the monitoring form, along with the blood bag or product, are returned to the blood bank by the patient |  |  |  |  |  |
|  | registration | completion of the transfusion reaction reporting form must be done within 24 hours with the coordination of the senior physician of Hemovigilance |  |  |  |  |  |
|  | registration | The adverse reaction form should be sent to the Blood Transfusion Organization within 48 hours |  |  |  |  |  |

**S table 6- "Returning the blood and blood products to the blood bank and disposing them"**

| activities | label | initial actions | second-level actions | Third-level actions | observations (Yes or No) |
| --- | --- | --- | --- | --- | --- |
| Disposal and return of the products from the unit to which the transfusion set was connected | support | It is destroyed with a special waste incinerator |  |  |  |
| If the transfusion set has not been connected to the blood bag or product | clinical | The labels of the returned blood bags and products are recorded on a separate form |  |  |  |
|  | registration | Completion of the blood and blood product non-utilization form on a monthly basis | Completion by blood bank personnel | Medical center |  |
|  | registration |  |  | Date of form completion |  |
|  | registration |  |  | Type of blood product |  |
|  | registration |  |  | Number (label) of the blood product |  |
|  | registration |  |  | Date of production |  |
|  | registration |  |  | Expiration date |  |
|  | registration |  |  | ABO/Rh |  |
|  | registration |  |  | Reason for non-utilization |  |
|  | registration |  |  | Returning to the blood transfusion organization for disposal as biological waste |  |
|  | registration |  |  | Disposing of as biological waste in medical centers |  |
|  | registration |  |  | Blood transfusion organization name |  |
|  | registration |  |  | Name and surname of the form completer |  |
|  | registration |  |  | hospital blood bank |  |
|  | registration |  | Completion by the recipient of the form | Name and surname of the form recipient |  |
|  | registration |  |  | Signature of the form recipient |  |
|  | registration |  |  | Date of form receipt by the recipient |  |
|  | support | Sending the form with the products to the blood transfusion organization |  |  |  |
